# Concentration of Microparticles Using Flexural Acoustic Wave in Sessile Droplets

**DOI:** 10.3390/s22031269

**Published:** 2022-02-08

**Authors:** Tao Peng, Luming Li, Mingyong Zhou, Fengze Jiang

**Affiliations:** 1 State Key Laboratory of High-Performance Complex Manufacturing, College of Mechanical and Electrical Engineering, Central South University, Changsha 410083, China; prettyage@foxmail.com (T.P.); lumingllm@csu.edu.cn (L.L.); zmy_csu@163.com (M.Z.); 2 Institute of Polymer Technology (LKT), Friedrich-Alexander-University Erlangen-Nurnberg, Am Weichselgarten 9, 91058 Erlangen, Germany

**Keywords:** particle concentration, flexural wave, acoustofluidic, numerical simulation

## Abstract

Acoustic manipulation of microparticles and cells has attracted growing interest in biomedical applications. In particular, the use of acoustic waves to concentrate particles plays an important role in enhancing the detection process by biosensors. Here, we demonstrated microparticle concentration within sessile droplets placed on the hydrophobic surface using the flexural wave. The design benefits from streaming flow induced by the Lamb wave propagated in the glass waveguide to manipulate particles in the droplets. Microparticles will be concentrated at the central area of the droplet adhesion plane based on the balance among the streaming drag force, gravity, and buoyancy at the operating frequency. We experimentally demonstrated the concentration of particles of various sizes and tumor cells. Using numerical simulation, we predicted the acoustic pressure and streaming flow pattern within the droplet and characterized the underlying physical mechanisms for particle motion. The design is more suitable for micron-sized particle preparation, and it can be valuable for various biological, chemical, and medical applications.

## 1. Introduction

Particle concentration at the microscale is of great significance in various biomedical and biochemical applications, including micro/nano-assembly [1,2], tissue engineering [3,4], and biomedical detection [5]. The concentration process is usually a crucial sample preparation step in related biomedical applications such as biosensors [6], and 3D cell culture [7]. Microparticle concentration realized by efficient fluidic methods has become an important research area. Many techniques based on various principles have been demonstrated to achieve particle concentration, such as magnetic [8,9], acoustic [10,11,12], optical [13], and dielectrophoretic [14] methods.

Among the various methods, particle concentration based on acoustofluidic manipulation has attracted wide attention due to its low-cost, high-sensitivity, label-free, and biocompatibility nature [15,16]. With acoustic wave actuation, precise and efficient particle manipulation from nanometer to micron scale can be achieved based on the induced acoustic radiation force and acoustic streaming [17]. Existing methods for acoustic concentration mainly include surface acoustic wave (SAW) [6,18,19], and flexural wave [12,20,21] vary with the used transducer. According to the analytical equation *f* = *c*/*λ*, SAW with the required resonant frequency *f* and specific type can be generated by fabricating interdigital electrodes (IDTs) with specific finger width on the substrate such as 128° YX Lithium Niobate [22,23]. Where *c* is the sound velocity in the substrate and *λ* is the wavelength. High frequency (~MHz) SAW often transport particles by generating streaming flow within the fluid [24,25]. Focused IDTs can achieve in-droplet particle concentration continuously by producing powerful vortex streaming [26]. Slanted IDTs enable broad-band frequency SAW excitation, and the vortex streaming induced in the droplet can be used for centrifugation to achieve the enrichment of nanoparticles [10]. For straight IDTs, four different regimes of particle concentration can be identified depending on the sessile droplet diameter [11]. Our group [6] has developed an acoustofluidic chip based on unidirectional surface acoustic wave centrifugation in droplets, which can quickly enrich the sub-micron particles.

SAW provides an effective way for micron and nanoparticle concentration. It usually uses lift-off and metal evaporation processes to fabricate interdigital electrodes on expensive piezo-substrates, such as Lithium Niobate and Lithium Tantalate. SAW can achieve more intense acoustic streaming and can be used for sub-micron and nano-particle manipulation [5,27]. In comparison, flexural wave-driven acoustophoretic devices utilize low frequency (kHz) inexpensive commercial buzzers to actuate glass and produce motion within the fluid region [21,28,29]. Compared with SAW chip, its production cost is less than $1 and the production process is easier. It has been successfully implemented in many lab-on-chip applications, such as micromixer [30], bioparticle isolation [31], and on-chip pumping [32]. Liu et al. [12] demonstrated using multi-well plates with integrating multiple flexural wave transducers to achieve particle and cell enrichment in sessile droplets. Kurashina et al. [33] demonstrated cell agglomeration in a 24-well plate using MHz-order acoustic wave. Whitehill et al. [34] evidenced experimentally the collection of microparticles at the solid-liquid interface in a sessile droplet due to a hydrodynamic focusing mechanism by low-frequency vibrations. Oberti et al. [35] demonstrated multiple patterns of particle gathering in sessile droplets driving by acoustic radiation force. Lei et al. [36] showed the interplay between streaming and radiation pressure in particle patterning. Riaud et al. [37] investigate the acoustic streaming in sessile droplets exposed to surface acoustic waves, and the results showed the precise streaming flows and transitions inside a drop subjected to ultrasonic waves.

Different from the existing publications, in this work, we studied the acoustic concentration of particles in multi-droplets on hydrophobic surfaces. Our work confirmed the simultaneous concentration of microparticles and cells in multiple droplets placed on the hydrophobic plate using only a single piezoelectric transducer with a simpler structure and high efficiency. The results obtained here should contribute to the understanding of related physical processes and provide theoretical guidance for the preparation of acoustofluidic concentration devices.

Here, we aim to develop an acoustofluidic chip with characteristics such as simplicity, low cost, and low power consumption for the efficient concentration of micron-scale particles. With these advantages, the proposed particle concentration method can be readily integrated with other on-chip biosensors to enhance the diagnosis process.

## 2. Materials and Methods

### 2.1. Working Principle

**Figure 1:**
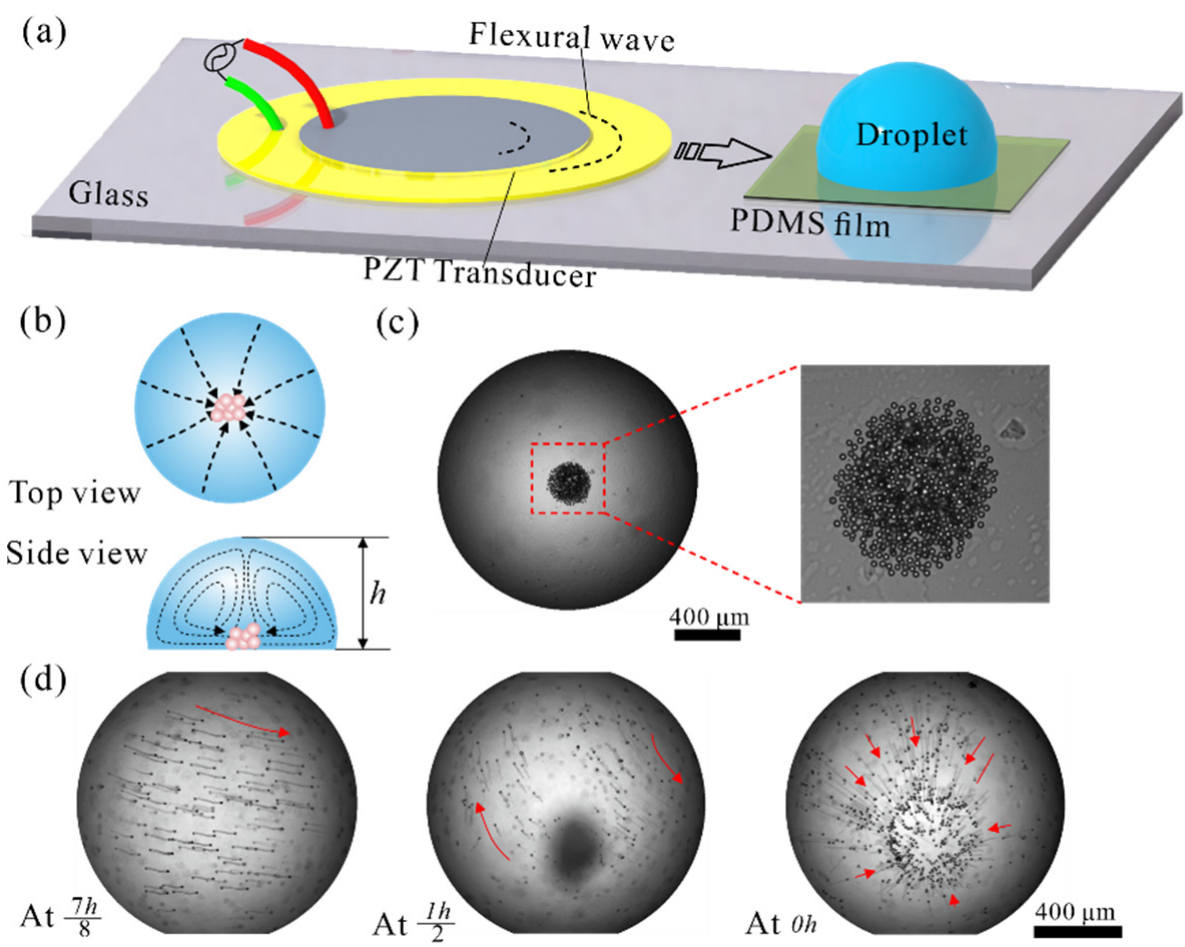
(**a**) Schematic presentation of the acoustofluidic concentration device. (**b**) Principle of the concentration process. (**c**) Experimentally observed 10 μm particle concentration at 48 kHz, 40 Vpp. (**d**) The trajectory of particles in cross-sections at different heights.

The device demonstrated here has a relatively simple structure compared with other acoustofluidic platforms. Figure 1a depicts the schematic image of the acoustofluidic concentration device, which consists of a 1 mm thick glass substrate, 25 μm thick Polydimethylsiloxane (PDMS) film, and a Pb-based lanthanum doped zirconate titanate (PZT) transducer. The flexural waves generated by the PZT transducer propagate and couple into droplets through the glass and PDMS film waveguide.

Figure 1b shows the principle of particle concentration. The normal actuation of the droplet by flexural waves induces two streaming vortexes in the droplet. The streaming induced drag force will cause the particles in the droplet to rotate with the vortex. Under the influence of streaming drag, gravity, and buoyancy force, Polystyrene (PS) particles gather toward the central area at the bottom plane. Figure 1c shows the result of 10 μm particle concentration. The randomly distributed 10 μm PS was gathered at the central area of the droplet.

### 2.2. Experimental Setup

Droplet-based fluidic chip has attracted widespread attention in the fields of biology and chemistry in recent years [38], as droplets provide a clean and stable experimental environment by acting as a separate reservoir [26]. In the experimental process, PZT transducer (7BB-20-6L0, Murata, Tokyo, Japan) was glued to the substrate. The PDMS film was bonded to the substrate employing surface activation through 70 s O_2_ plasma treatment and heated at 80 °C for 2 h to enhance bonding effect. As shown in Figure S1, the untreated PDMS surface owns hydrophobic properties compared to the hydrophilic glass surface. The water contact angle is about 105°, which helps the droplets maintain equilibrium shape with regular circular contour without rolling and make the experimental process more controllable [39,40]. The ambient temperature during the experiment is 20 °C, and the relative humidity is 80%.

The signal generator (Tektronix, AFG 31102) was adopted to provide alternating current (AC) electric signals. The AC signals were amplified by a power amplifier (Dongrun, FPA1013, Yantai, China) and then supplied to the PZT transducer. A high-speed camera (AcutEye 4.0, Rocketech, Changsha, China) and an inverted fluorescence microscope (Ti2-U, Nikon, Tokyo, Japan) were used to observe and record the experimental process. PS microspheres were diluted in deionized water and used for the experiments. The droplet was placed on the surface of PDMS by pipette with a volume of about 3 μL. Liver cancer cells were obtained by culturing cell lines.

### 2.3. Numerical Modeling Setup

As shown in Figure 1d, by adjusting the microscope focus, we obtained images of different cross-sections in the droplet. At the height of 7 h/8 h, the particles exhibit a downward rotational movement. At the height of 0.5 h, the particles rotate toward two opposite directions. At the bottom area, the particles move linearly to the concentrated area under the streaming drag force. These results show that acoustic streaming is the main reason for transporting particles and realizing concentration in the droplet. Based on this, we performed a series of numerical simulations implemented by the commercial finite element (FE) software COMSOL Multiphysics (v5.4, COMSOL, Stockholm, Sweden) to explore the underlying physics. The numerical model setup refers to the existing literature used to predict particle motion and acoustic field under flexural wave actuation in a continuous microfluidic system, which has been experimentally verified and shows good consistency compared with the experimental observation [29]. It has been proven that the resonance frequencies of 2D and 3D flexural acoustic wave systems are almost the same. Therefore, a 2D model was chosen to study the related mechanism to simplify the calculation.

**Figure 2:**
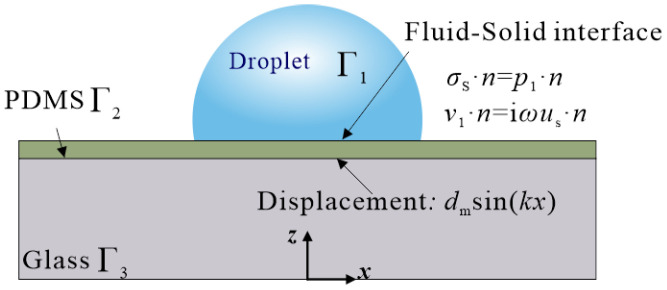
The numerical model details. In the FE model, Γ_1_ is the droplet area, Γ_2_ is the PDMS area, and Γ_3_ is the glass area.

The detail of the numerical model is shown in Figure 2. The acoustic pressure distribution in the droplet is related to vibration mode w(x,z) at the contact interface. Instead of solving the entire model, a displacement boundary condition was applied. The droplet was defined as a sphere with a diameter of 1.8 mm and a central angle of 210°.

In the FE software, the ‘thermoviscous acoustic–solid interaction’ module was used to solve the droplet’s first-order acoustic pressure and velocity distribution. The outer surface of the droplet was set as the impedance boundary. The upper surface of the glass was set as displacement boundary with *d*_m_sin(*kx*), where *k* = *ω*/*c* is the acoustic wavenumber and *d*_m_ is the amplitude. The value of *d*_m_ ranges from1–30 nm in the numerical simulation. The side of the glass was set as the low-reflection boundary. In the previous studies of particle concentration in the pipeline, the simulation ignores acoustic streaming due to their weak effect on particle motion [41,42]. In this study, acoustic streaming is the primary cause of particle concentration. The ‘laminar flow’ module was used to solve acoustic streaming based on the stress method and a stationary solver [43]. The depth of the viscous penetration layer was calculated by $\delta =\sqrt{\mu /\pi \rho_{0}f}$ [44], and the calculated *δ* = 2.56 μm, where *μ* is the dynamic viscosity, and *ρ*_0_ is the liquid density. The ‘particle tracing’ module was used to predict particle trajectory in the droplet. Newton’s second law governed the motion of the particles:

(1) $$m_{p}\frac{dv_{p}}{dt}=F_{D}+F_{G}+F_{B}$$

where *m*_p_ is the particle mass, *F*_G_ is gravity, and *F*_B_ is buoyancy. The drag force *F*_D_ resulting from acoustic streaming was estimated via the celebrated Stokes equation $F_{D}=6\pi \mu a(\langle v_{2}\rangle -v_{p})$, where *a* is particle radius, <***v***_2_> is the time-averaged streaming velocity, and ***v***_p_ is particle velocity. The buoyancy *F*_B_ was estimated by $F_{B}=4\pi \rho_{0}ga^{3}/3$.

The amplitude and distribution of acoustic radiation force were calculated to determine the influence of streaming drag force and acoustic radiation force on particle motion. The acoustic radiation force *F*_r_ results from the transfer of momentum from an acoustic field in an attenuating medium and it was calculated with the first-order acoustic field [43]:

(2) Fr=−πa3[2κ03Re[f1*p1*∇p1]−ρ0Re[f2*v1*∇v1]]

where *a* is particle radius, *κ*_0_ is liquid compressibility, *ρ*_p_ is the particle density, *p*_1_ is the first-order acoustic pressure, *v*_1_ is the acoustic velocity. Re(A) is referred to the real part of a complex variable. The asterisk denotes the complex conjugate of the quantity.

The compressibility factor *f*_1_ and density *f*_2_ are given by:

(3) $$f_{1}=1-\tilde{\kappa },\begin{array}{cc} & with\begin{array}{cc} \tilde{\kappa }=\frac{\kappa_{p}}{\kappa_{0}} & \end{array} \end{array}$$

(4) $$f_{2}=\frac{2(1-\Gamma )(\rho_{p}-\rho_{0})}{2\rho_{p}+\rho_{0}(1-3\Gamma )}$$

(5) $$\Gamma =-\frac{3}{2}[1+i(1+\frac{\delta }{a})]\frac{\delta }{a}$$

where *κ*_p_ is particle compressibility, $\tilde{\kappa }$ is the relative compressibility, Γ is a pre-defined factor, for 10 μm particle the numerical value is −0.384–0.482i. The above Equations (2)–(5) were used to estimate the acoustic radiation force.

The mesh was adjusted to capture the physics inside the boundary layers. The free triangular mesh was adopted for the calculation. In the fluid domain (droplet), the computational mesh is generated from a maximum element size 0.2 *δ* at the fluid boundary. The maximum element size in the bulk of the fluid and solid was given by 3 *δ*.

## 3. Results and Discussions

### 3.1. Numerical Simulation Results

**Figure 3:**
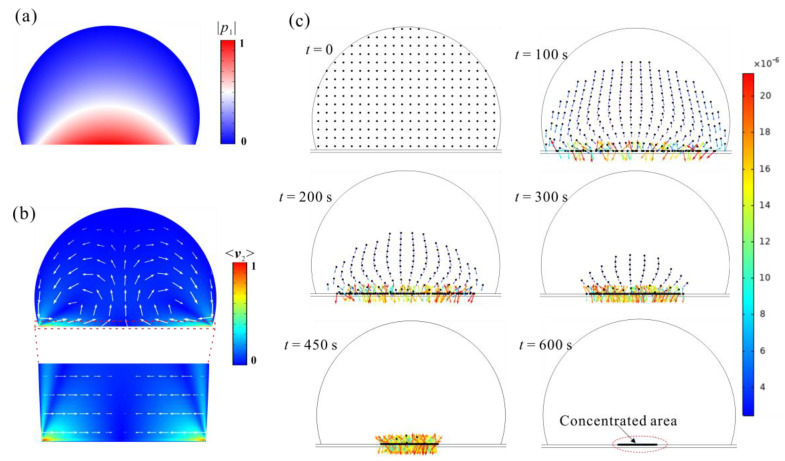
(**a**) The normalized acoustic pressure distribution. (**b**)The normalized acoustic streaming, the arrows in the primary and enlarged area are <***v***_2_> and <***v***_2x_>. (**c**) The time-series distribution of 10 μm particles upon *d*_m_ = 10 nm. The arrow represents the velocity direction, and the color represents the magnitude of velocity, m/s.

Based on the above described numerical model, we performed simulations for the acoustofluidic device. The frequency used in the simulation was 48 kHz which is consistent with the experimental process. Figure 3a shows the normalized acoustic pressure field. By adjusting the frequency from 40–50 kHz, we found that the acoustic pressure distribution does not change much, so the influence of frequencies on particle concentration was ignored.

Based on the calculated first-order acoustic field, we estimated the acoustic radiation force received by the particles through Equations (2)–(5). Figure S2 shows the distribution of acoustic radiation force received by 10 μm particles resulting from the scattered waves, and the maximum appears in the area near the bottom and the sidewall. In the bulk area of the droplet, the acoustic radiation force is less than 10^−14^ N. Figure 3b demonstrated the acoustic streaming distribution. Two vortices form in the droplet, which causes the transportation of particles. The maximum velocity appears in the area near the bottom of the droplet boundary.

**Figure 4:**
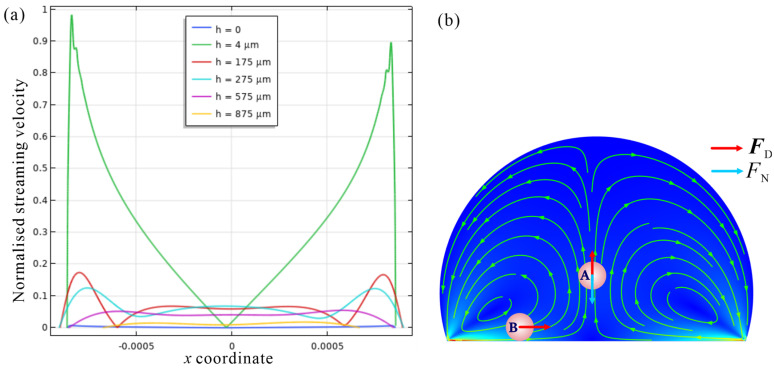
(**a**) The intensity of acoustic streaming in the droplet of different cross-sections. The height of the droplet is 1.14 mm. (**b**) The force conditions for particles to gather in two positions.

As shown in Figure S3, the acoustic radiation force is negligible compared with streaming drag force, as is much weaker in the cross-sections at different heights. Therefore, it can be considered that acoustic streaming is the main propulsion for inducing particle concentration. As shown in Figure 3c, with the increase of time, the initially randomly scattered 10 μm particles move to the bottom plane under the action of net downward force and accelerate to the concentrated area (Also seen in Supplemental Movie S1). The reason is that the streaming intensity is stronger near the bottom than other locations, as shown in Figure 4a.

From the simulation results, it is concluded that the formation of concentration phenomenon needs to satisfy two conditions: the drag force in the concentrated area is less than the net gravity, for which condition the particles will not be lifted by the drag force, as shown in location A in Figure 4b; the acoustic streaming is also necessary to ensure that particles can be transported to the concentration area at the bottom, as shown in location B in Figure 4b.

Since the PS particle density of *ρ*_p_ = 1050 kg/m^3^ is greater than the liquid of *ρ*_0_ = 1000 kg/m^3^. For a specific size PS, the net downward force produced by gravity and buoyancy is $F_{N}=\frac{4\pi a^{3}g(\rho_{p}-\rho_{0})}{3}$, which is constant and the direction is downward. For 10 μm particles, the value is 0.257 pN. To ensure that the particles will not be lifted at the bottom, the following analytical equation needs to be met:

(6) $$\frac{F_{N}}{F_{D}}=\frac{2a^{2}g(\rho_{p}-\rho_{0})}{9\mu (\langle v_{2}\rangle -v_{p})}>1$$

where g is the gravitational acceleration.

It can be seen from Equation (6) that under a certain streaming velocity, it is easier for larger-size particles to meet Equation (6), as Equation (6) is proportional to *a*^2^. The equation can be used to determine the minimum acoustic streaming velocity at which particles of a specific size can be concentrated, thereby determining the chip’s input power. Under a certain streaming velocity, this equation can also be used to solve the inequality to determine the threshold value for a minimum size that is satisfied to achieve particle concentration.

For smaller-sized particles, weakening the acoustic streaming by reducing the input voltage can also meet this condition. However, with *F*_N_ dominating the particles’ motion towards the bottom plane, it takes a longer time for the particles to be transported to the concentrated area. We also verified this through simulation. When *d*_m_ = 1 nm, the 10 μm particles fall to the bottom surface under the dominant *F*_N_. Due to the weak streaming velocity, the particles are still randomly scattered on the bottom surface at 600 s, as shown in Figure S4 and Supplemental Movie S2.

By increasing the input voltage to enhance the streaming strength, the concentration process could be accelerated. However, more robust streaming will cause the particles to rotate with the vortex, which will be adverse for concentration. As shown in Figure S5, when *d*_m_ = 30 nm, the 10 μm particles will rotate due to the strong streaming effect, and less obvious concentration forms (Supplemental Movie S3).

We also verified from the simulation that when the phase of the acoustic wave changes, the position of the concentrated area also changes slightly. However, the total effect of particle concentration will not be affected as shown in Figure S6.

### 3.2. Experimentally Observed Particle Concentration Process

In the experimental process, by sweeping several frequencies experimentally spans from 35 to 50 kHz, we found that the maximum streaming velocity appeared at 48 kHz, so 48 kHz was chosen as the resonance frequency for studying the particle concentration process. The resonance denotes the peak streaming response in the frequency response. This is most likely caused by damping from the waveguide and external connections and is typically in a damped resonance system.

**Figure 5:**
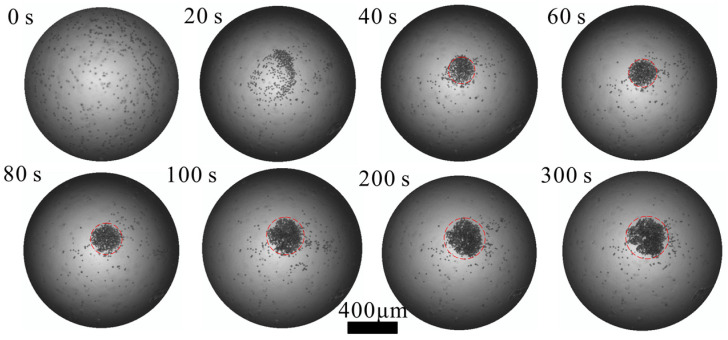
Time-series images of 10 μm particle concentration process. (Supplemental Movie S4) The images were taken at the *h* = 0. The equivalent diameter of the concentrated region at 40, 60, 80, 100, 200, 300 s are 216, 228, 265, 306, 340, 356 μm respectively.

As shown in Figure 5, at 0 s, without an acoustic field, the particles are randomly scattered in the droplets. At a driving amplitude of 30 Vpp, 10 um PS concentrate at the center within 40 s. As exposure to the acoustic field increases, the concentrated effect becomes more evident, and the number of particles in the concentration area increases.

As the number of unaggregated particles continues to decrease, the concentration effect weakens. To count the number of enriched particles or cells, fluorescent dyes can be used to stain the particles or cells before the concentration process. The fluorescence intensity can characterize the number of particles in the concentration area as the fluorescence intensity is positively correlated with the number of particles. This is consistent with simulation results.

### 3.3. Concentration of Microparticles with Different Sizes

**Figure 6:**
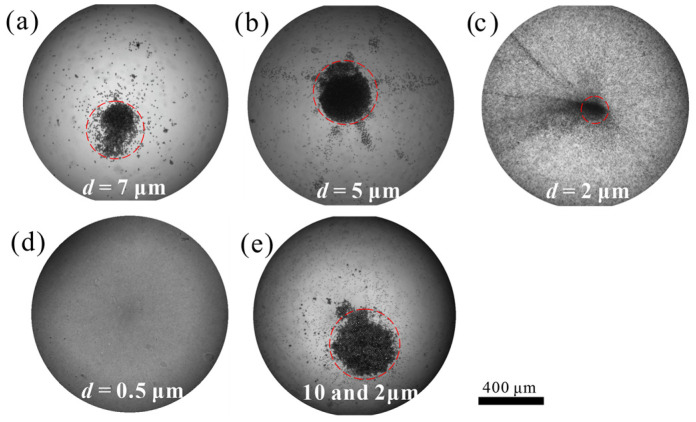
Particle concentration of different sizes within 300 s (48 kHz, 40 Vpp). (**a**–**d**) 7, 5, 2, and 0.5 μm particle. Where *d* is particle diameter. (**e**) 10 and 2 μm mixed particles. The images were taken at the *h* = 0. The equivalent diameter of the concentrated region for (**a**–**c**,**e**) are 352, 391, 167, and 429 respectively.

Based on the proposed acoustofluidic concentration device, we further studied the concentration effect of particles of different sizes through experiments. As shown in Figure 6a,b, both 7 μm and 5 μm particles can be concentrated with the chip. For 2 μm PS particles, due to the relatively small weight, it is easier to be lifted by acoustic streaming and the concentration effect becomes worse.

For 0.5 μm particles, concentration cannot be achieved. When concentrating mixed particles of 10 and 2 μm, larger-sized particles are easier to concentrate than smaller-sized particles. These results demonstrate that the concentration mechanism is related to particle size, and larger-sized particles are easier to be concentrated. Due to the difference in the droplet’s position on the PDMS surface during the experiment, the phase difference causes the concentrated area to shift slightly but does not affect the total concentration effect (Figure 6a,b). The concentrated area is always close to the droplet center due to the waveform. These results are well consistent with analytical estimates and numerical calculation results.

### 3.4. Simultaneously Concentration of Microparticles in Multiple Sessile Droplets

**Figure 7:**
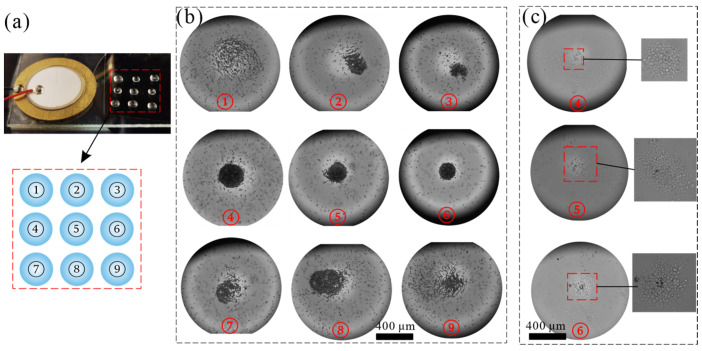
Simultaneously concentration of 10 μm particles and cells in multiple sessile droplets. (**a**) Photograph image of the chip and numbered diagram of droplets. (**b**) The effect of particle concentration in nine droplets at 200 s. (**c**) Concentration effect of liver cancer cells in three droplets.

Then, we demonstrated simultaneous particle concentration in multiple droplets. As shown in Figure 7a, nine droplets containing microparticles were pipetted on the working area. As shown in Figure 7b, the PS particles in the nine droplets all show the behavior of gathering towards the central area. However, the gathering location differs due to the phase difference caused by the droplet’s position, and the concentration effect also differs due to the wave propagation and attenuation. The concentration effect in droplets 4–6 is better than in the other six locations. When the time droplet being stimulated by acoustic wave increases to 300 s, a suitable concentration effect in droplets 1–3 and 6–9 can also be observed.

Based on the PS particles concentration results, we then achieved simultaneous concentration of liver cancer cells HepG2 (about 12 μm diameter) in locations 4–6 (Figure 7c). These experimental results [e.g., Figure 7] indicate the potential of the chip system for the application of multicellular spheroids and 3D cell culture [7].

We further investigated the state change of a 3 μLdroplet on PDMS film over time. As shown in Figure S7, within 10 min, the volume reduction and the profile change of droplet can be negligible. This means that droplet evaporation will not affect the particle concentration process. The volume of droplet evaporation increases over time. At 50 min, the droplet volume reduced more than 30%, and its profile has changed a lot compared with its initial state. In the experimental process, we also found that when driven by a commercial buzzer, although the input power is high (~40 Vpp), it did not cause a significant temperature rise (the rise did not exceed 5 °C). The reason may be that when the adopted frequency is far from the resonance frequency of the buzzer, the energy conversion efficiency is relatively low. Once the temperature rise is high, a real-time refrigeration device can be designed using the commercial semiconductor Peltier cooler. The relationship between acoustic streaming patterns with droplet profile and volume still needs to be explored in the future.

## 4. Conclusions

In conclusion, we explored the potential of concentrating particles in sessile droplets benefiting from flexural acoustic waves. The proposed approach uses a simple fabrication process where a glass slide is utilized as the substrate for flexural wave propagation and a PDMS film is utilized for placing droplets. The design does not rely on the precise positioning of the droplet due to the relatively large acoustic wavelength. Through experiments and numerical simulations, we demonstrated the concentration process and explained the mechanism. The experimental results confirmed the effective concentration of micron-scale particles and tumor cells. Analytical and numerical results show that the concentration of particles is mainly affected by acoustic streaming, and it is necessary to ensure that the drag force does not lift the particles. The relative magnitude of the net gravity and the streaming drag force determines whether the particles can be concentrated. The motion of microparticles suspended in a droplet actuated by flexural acoustic waves depending on the microparticle diameter, the wave attenuation length, and the frequency of waves still needs further study. We expect the proposed design to serve as a promising tool for biosample preparation tissue engineering and micro-fabrication applications with its simplicity, noncontact, and stability.

## Supplementary Materials

The following supporting information can be downloaded at: https://www.mdpi.com/article/10.3390/s22031269/s1, Figure S1: The water contact angle of glass and PDMS film; Figure S2: The acoustic radiation force distribution in the droplet at dm = 10 nm. The arrow represents the direction of acoustic radiation force, Figure S3: (a) Acoustic radiation force and (b) streaming drag force at different cross-sections when the amplitude is 10 nm, Figure S4: When the amplitude is 1 nm, the 10 μm particle distribution changes over time, Figure S5: When the amplitude is 30 nm, the 10 μm particle distribution changes over time, Figure S6: The particle concentration with the changing in wave phase at dm = 10 nm and f = 48 kHz. The background represents the acoustic pressure field, and the streamline represents the streaming flow, Figure S7: The state change of a 3μLdroplet on PDMS film and the red line represents the initial droplet profile.
