# Supplementary material for: Concentration of Microparticles Using Flexural Acoustic Wave in Sessile Droplets
Source: Sensors (Basel). 2022 Feb 8;22(3):1269. doi: 10.3390/s22031269 (PMC8839499; doi:10.3390/s22031269)
Supplement: Supplementary file 1 [file sensors-22-01269-s001.zip › sensors-1565672-supplementary.pdf]

Supplemental Information

Concentration of Microparticles Using Flexural Acoustic Wave in Sessile Droplets

Tao Peng [1], Luming Li [1], Mingyong Zhou [1] and Fengze Jiang [2,*]

[1] State Key Laboratory of High-Performance Complex Manufacturing, College of Mechanical and Electrical Engineering, Central South University, Changsha 410083, China; 183701023@csu.edu.cn (T.P.); luminglIm@csu.edu.cn (L.L.); zmy_csu@163.com (M.Z.)

[2] Institute of Polymer Technology (LKT), Friedrich-Alexander-University Erlangen-Nurnberg,
Am Weichselgarten 9, 91058, Erlangen , Germany

* Correspondence: Author: fengze.jiang@fau.de

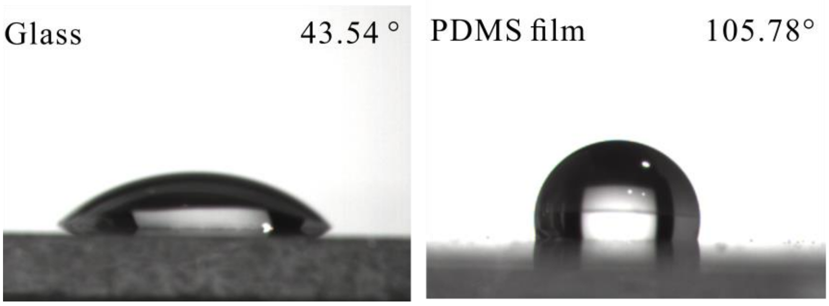

Figure S1. The water contact angle of glass and PDMS film.

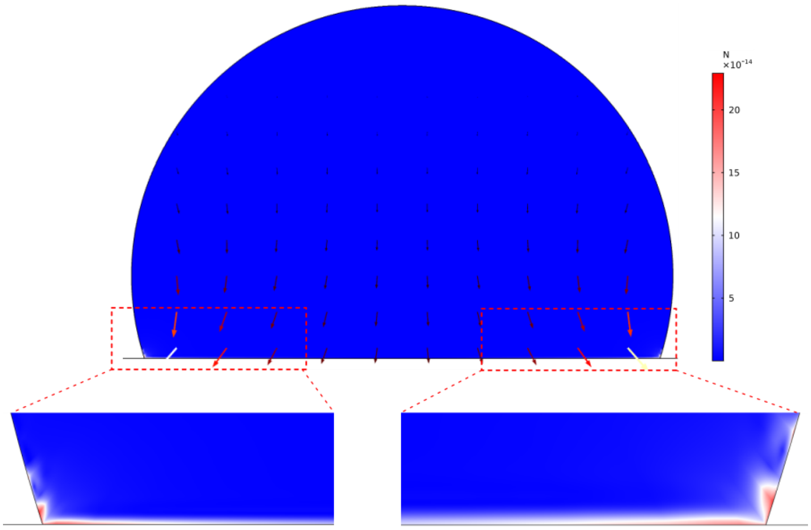

Figure S2. The acoustic radiation force distribution in the droplet at d_m = 10 nm. The arrow represents the direction of acoustic radiation force.

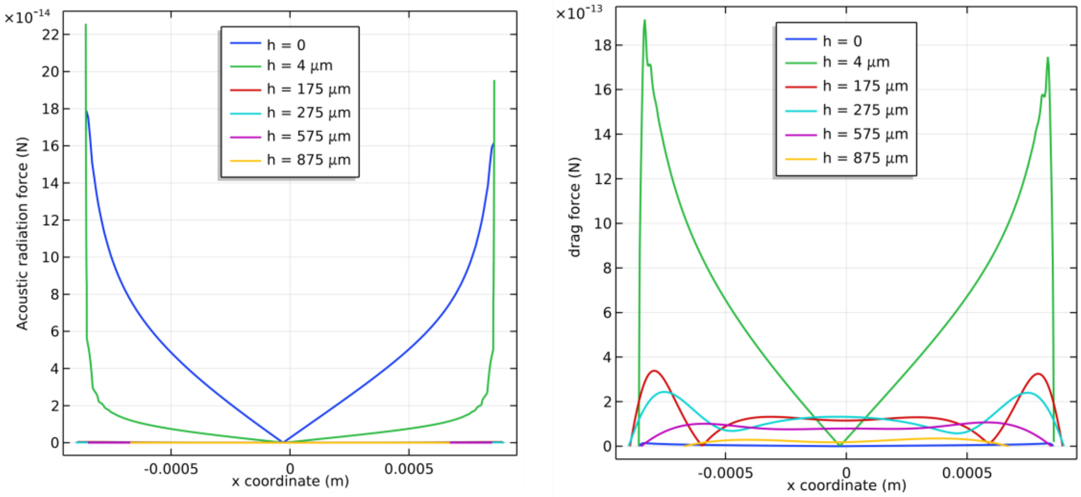

Figure S3. (a) Acoustic radiation force and (b) streaming drag force at different cross-sections when the amplitude is 10 nm.

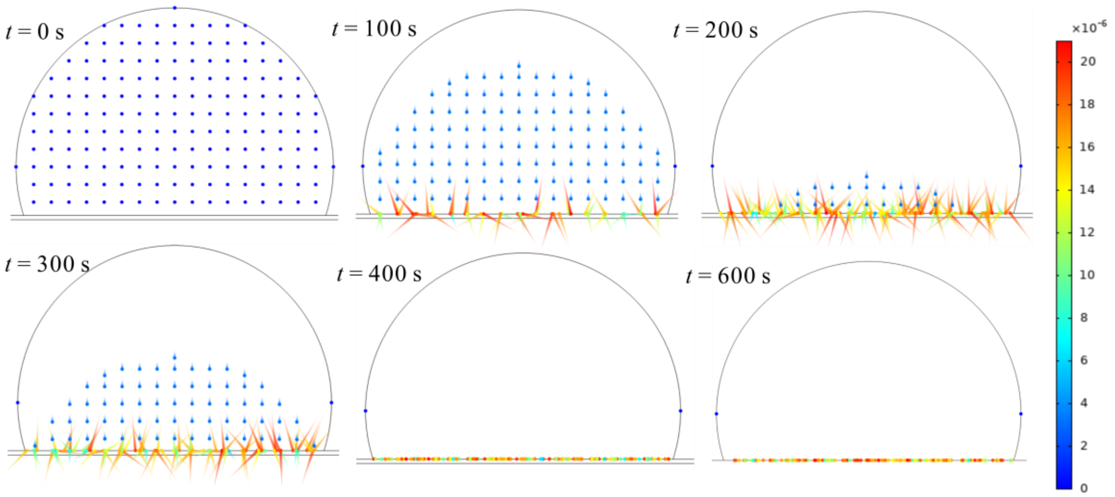

Figure S4. When the amplitude is 1nm, the 10 μm particle distribution changes over time.

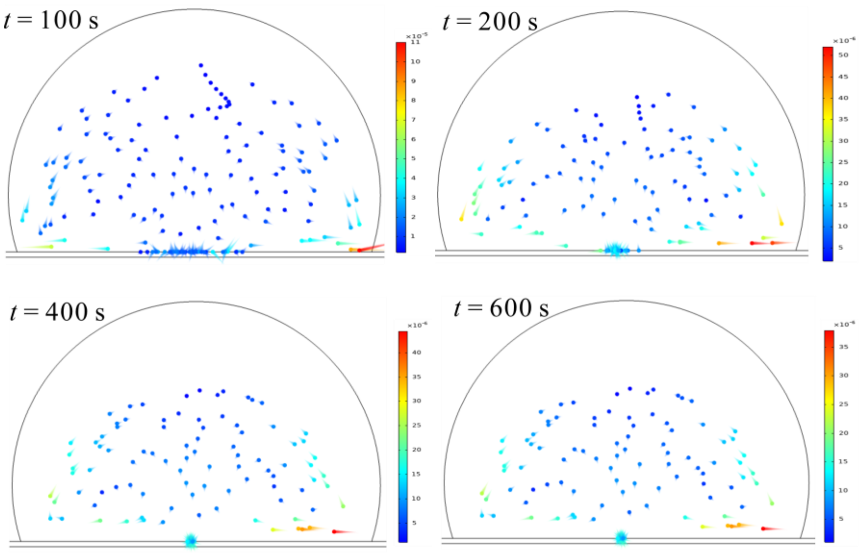

Figure S5. When the amplitude is 30 nm, the 10 μm particle distribution changes over time.

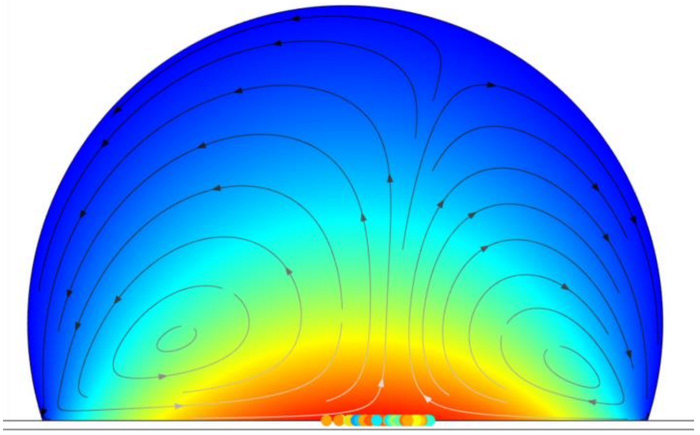

Figure S6. The particle concentration with the changing in wave phase at $d_m = 10$ nm and $f = 48$ kHz. The background represents the acoustic pressure field, and the streamline represents the streaming flow.

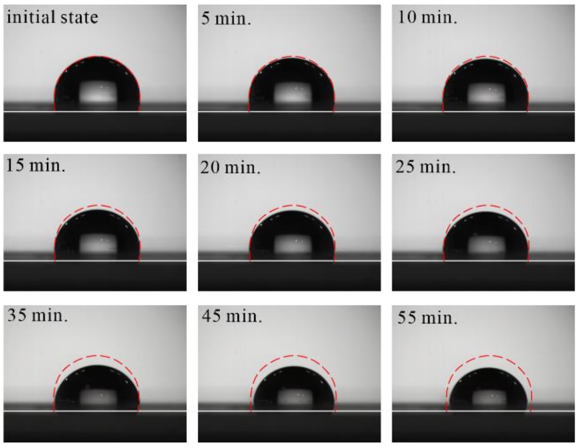

Figure S7. The state change of a 3μLdroplet on PDMS film and the red line represents the initial droplet profile
